# Editorial: Brain Activity Patterns During Dreams

**DOI:** 10.3389/fnins.2022.802778

**Published:** 2022-05-10

**Authors:** Wei Wang, Roger C. Ho

**Affiliations:** ^1^Department of Psychology, Norwegian University of Science and Technology, Trondheim, Norway; ^2^Department of Psychological Medicine, National University of Singapore, Singapore, Singapore

**Keywords:** Editorial, dream, cerebral, clinical, basic research

Together with Dr. Roger Ho, I would like to thank the 21 authors, 12 reviewers, and frontiersin.org who worked with us for the Research Topic (RT) “*Brain Activity Patterns During Dreams*” for the journal “Frontiers in Neuroscience.” The RT was mainly complied within the year 2020, which was the most difficult time as it fell during the COVID-19 pandemic period. As we all know, fighting against COVID-19 is a challenge for all of us, especially for our authors and reviewers.

Nevertheless, when the RT was launching, we successively received six manuscripts. We agreed on publishing four of them since we had to stick to the standards of the journal and the scientific publication criteria. We are very grateful to all endeavors from all our crew members.

Although the number of papers is small, authors have presented a broad area of cerebral activities during dreaming. The four Research Topics ranged from normal sleep physiology to clinical sleep disorders and from integrated brain signals to neuroimaging techniques. From a limited angle, these papers have described brain activities regarding dreaming. Even though these lab investigations, literature reviews, and opinion expressions are based on on-the-spot observations, they are the result of ongoing work representing the neural bases during dreaming. As Ruby has stated, the neural bases of dreaming are still unclear instead of being fully elucidated. This opinion also acts as a further call for discovering more brain working patterns underlying dreaming in coming years.

We are also glad to see that these four papers have received great early notice with excellent Altimetric scores and by other scientific evaluations. Both Roger and I are very clear-minded and we now look forward to seeing the further progression in this area, and we are very confident that our wishes will be accomplished soon.

## Author Contributions

Both authors listed have made a substantial, direct, and intellectual contribution to the work and approved it for publication.

## Conflict of Interest

The authors declare that the research was conducted in the absence of any commercial or financial relationships that could be construed as a potential conflict of interest.

## Publisher's Note

All claims expressed in this article are solely those of the authors and do not necessarily represent those of their affiliated organizations, or those of the publisher, the editors and the reviewers. Any product that may be evaluated in this article, or claim that may be made by its manufacturer, is not guaranteed or endorsed by the publisher.

